# Effect of reagents used during detection and quantification of *Ascaris suum* in environmental samples on egg viability

**DOI:** 10.2166/wst.2017.324

**Published:** 2017-07-11

**Authors:** Isaac Dennis Amoah, Poovendhree Reddy, Thor Axel Stenström

**Affiliations:** 1 **Isaac Dennis Amoah** (corresponding author) **Thor Axel Stenström** Institute for Water and Wastewater Technology, Durban University of Technology, P.O. Box 1334, Durban 4000, South Africa; 2 **Isaac Dennis Amoah Poovendhree Reddy** Department of Community Health Studies, Faculty of Health Sciences, Durban University of Technology, P.O. Box 1334, Durban 4000, South Africa

**Keywords:** *Ascaris suum*, inactivation, sludge, soil-transmitted helminths, viability, wastewater

## Abstract

Soil-transmitted helminths (STHs) are a major health concern globally. Infection is mostly through contact with contaminated water, food or soil. Therefore to break the cycle of viable transmission STH eggs must be quantitatively detected in the environment. The effect of different reagents on the viability of *Ascaris suum* eggs during laboratory detection and quantification was assessed and different incubation solutions compared. Sulphuric acid gave a slightly higher recovery percentage of viable eggs (91.2%) than distilled water (90.0%) and 0.5% formalin (87.6%), although the difference was not statistically significant (*p* > 0.05). Acetoacetic acid, ethyl acetate, ammonium bicarbonate, zinc sulphate, magnesium sulphate and Tween 80, are reagents widely used in test protocols for the detection and quantification of STH eggs. Eggs were exposed to these reagents for different time durations. Acetoacetic acid resulted in the highest loss of viability (3.4 ± 0.7% viable), while magnesium sulphate resulted in the least effect (88.5 ± 1.2% viable). In conclusion the use of the selected reagents in the detection of these eggs was found to affect the viability of exposed eggs, especially during prolonged exposures. Therefore we recommended that eggs be exposed for ≤ 5 minutes, to reduce the risk of viability loss.

## INTRODUCTION

In soil-transmitted helminth (STH) infection endemic regions it is estimated that wastewater may contain up to ∼3,000/L (Kamizoulis 2008; Mara & Sleigh 2010) of STH eggs, while documented counts in faecal sludge from public toilets are between 2,500 and 60,000 eggs/L (YenPhi et al. 2010). From septic tanks the concentrations are reported to be from 600 to 16,000 eggs/L (Yen-Phi, *et al*. 2010). Trönnberg *et al*. (2010) assessed the concentrations of STHs in the faecal vaults of urine-diverting (UD) toilets in the KwaZulu Natal Province of South Africa. Counts varied from below detection limit to a maximum of 1,425 eggs per gram (EPG) of faeces for *Ascaris lumbricoides,* 147 EPG for Trichuris trichiura and 703 EPG for Taenia spp. These STH eggs can survive for a long period of time in the environment, where fertilized eggs of *Ascaris* spp. for instance have been reported to survive for up to 7 years under favourable temperatures of 25 °C and humidity greater than 55% (WHO 2015). Due to the highly varying numbers, the detection and quantification of STH eggs is important for the determination of health risk due to exposure during the collection, conveyance, treatment and possible reuse of faecal waste. It is also important in the validation of wastewater treatment technologies (Peterson *et al*. 2011).

Ingestion of fertilized eggs of STHs may result in asymptomatic diarrhoea (Brownell & Nelson 2006). The most common STH infection is ascariasis, infecting 771.7–891.6 million people worldwide (Strunz *et al*. 2014), with the majority of these infections occurring in tropical and subtropical countries (Stolk *et al*. 2016). Although infection with STHs is not lethal it may result in impairment of physical and cognitive development (Naidoo *et al*. 2016).

The greatest risk to public health in relation to STH infections is the reuse of wastewater, sludge, compost etc. in agriculture (Habbari *et al*. 1999; Amahmid & Bouhoum 2005; Ensink *et al*. 2005). To ensure public safety several international and national agencies have recommended guidelines for wastewater reuse in agriculture (USEPA 1992; WHO 2006). These guidelines have been adopted by various national and regional bodies, such as the Union of National Associations of Water Suppliers and Waste Water Services from countries within the EU (Angelakis & Bontoux 2001). The guidelines account for not only the quality for reuse but also the treatment steps needed to achieve the desired quality. The World Health Organization (WHO 2006) recommends ≤1 helminth egg per litre for unrestricted agriculture as against no detectable helminth egg per litre of irrigation water by the US Environmental Protection Agency (USEPA 1992). To determine the suitability of wastewater or sludge etc. for reuse or the validation of treatment technologies there is the need for accurate determination of viable STH eggs in the various samples (wastewater, sludge etc.). However, there are a variety of methods, with the major ones being the United States Environmental Protection Agency (USEPA) (Schwartzbrod 1998) and the World Health Organization (WHO) methods (Ayres & Mara 1996), or variants of these (Amoah *et al*. 2017).

The variety in the techniques used is influenced by several factors, with key among these being the variability in the sample matrices, reagents available and personal preferences of the researcher (Mes 2003). Sludge, compost and UD wastes are often blended with a detergent solution, which could be Tween 20 or Tween 80, ammonium bicarbonate or others with the intention to increase the separation of the egg from solid materials in the samples (Zenner *et al*. 2002; Trönnberg *et al*. 2010). In addition to these detergents, there are several other reagents used with different purposes. For instance flotation solutions, such as zinc sulphate, magnesium sulphate and sodium chloride, are used to further separate the eggs from other particulate matter based on differential flotation of these materials (Maya *et al*. 2006; Rosa Xavier *et al*. 2010). A phase extraction step is included in some of the methods for the removal of lipid-soluble and ether-absorbing material from the samples (Rude *et al*. 1987; Nelson & Darby 2001). Diethyl ether, ethyl acetate and acetoacetic buffer are commonly used for this purpose.

This variety of reagents used may inadvertently affect the viability of the STH eggs. Long-term exposure to ZnSO_4_ has been shown to be toxic to STH eggs (Gaspard *et al*. 1996); in addition, soaking eggs in MgSO_4_ overnight may also inactivate embryonated eggs (Smith 1991). One per cent of formalin is mainly used as incubation solution for STH eggs but Oksanen *et al*. (1990) stated that it retarded their development as compared to water or 0.1 N H_2_SO_4_ (Nelson & Darby 2001). Phase extraction with acid-alcohol and diethyl ether also decreases egg viability (Nelson & Darby 2001). Inactivation of eggs may be due to the damage of the egg shell of the parasites, which protect the egg and enhance the survival ability of STHs. A change in the permeability of the cell-wall layer of eggs may result in the loss of viability (de Souza *et al*. 2001).

Despite the perceived effect of different chemicals/ reagents on the viability of helminth eggs, few quantitative assessments of the effects of the various reagents have been made. As part of an ongoing project aimed at developing a uniform method for the detection and quantification of STH eggs in environmental samples, a series of experiments were carried out to determine the effect of selected reagents (commonly used) on the viability of STH eggs, using Ascaris suum eggs as a surrogate.

## METHODOLOGY

### Preparation of stock solution

Ascaris suum eggs were purchased from Excelsior Sentinel Inc. (Ithaca, NY, USA). The eggs were collected from the intestinal contents of infected pigs and concentrated through a series of sieves. A working egg solution with approximately 1,000 eggs/mL was prepared by suspending the eggs in distilled water. Stock solution of eggs was stored at 4 °C to prevent any egg development. Individual samples were prepared by placing 1.0 mL of the working solution in 50-mL centrifuge tubes.

### Initial screening and comparison of incubation solutions

Viability of the eggs was determined, after initial screening, to ascertain their stage of development. Initial viability was determined by incubation in three different solutions: distilled water, 0.5% formalin and 0.1 N sulphuric acid. Eggs were viewed microscopically (×40) after 28 days of incubation and categorized as potentially viable or non-viable based on morphology. Undeveloped (at different cell stages) and embryonated eggs with a visible motile larva were considered potentially viable. Eggs with broken egg shell, visible internal globules and necrotic or immotile larvae were considered to be potentially non-viable (Moodley *et al*. 2008). Percentage viability was calculated for each incubation solution by dividing viable eggs by the total eggs counted and multiplying by 100, based on a minimum of 200 eggs counted per each microscopic reading. The solution that gave the highest percentage viability was then selected for the inactivation tests (next section).

### Inactivation tests

Acetoacetic acid (Sigma Aldrich, Germany), ethyl acetate (ACS reagent,≥ 99.5%; Sigma Aldrich, Germany), ammonium bicarbonate (ACS reagent, ≥99.5%; Sigma Aldrich, Germany), zinc sulphate (99.9%; Promark Chemicals, South Africa), magnesium sulphate ≥(99.0%; Promark Chemicals, South Africa), Tween 80 (Promark Chemicals, South Africa) and a combination of acetoacetic acid and ethyl acetate as well as a combination of acetoacetic acid and formalin were the reagents and combinations selected for the experiments. These were chosen based on their widespread use in several methods for the detection and quantification of STH eggs in environmental samples (Bornay-Llinares *et al*. 2006; Horiuchi *et al*. 2013; Fuhrimann *et al*. 2015; Rocha *et al*. 2016; Verbyla et al., 2016). The concentrations of these reagents were the same as recommended by the various methods. These are 100% for acetoacetic acid and ethyl acetate, 0.5% formalin, zinc sulphate of specific gravity of 1.3 (56.8%), saturated ammonium bicarbonate at 11.9% and 0.1% of Tween 80. These concentrations were not varied, so as to determine their effect on A. suum viability based on the recommended concentrations in the methods.

Approximately 1,000 A. suum eggs were exposed in 20 mL of each reagent for 5, 10, 30, 60, 90 and 120 minutes (each treatment was in triplicate). After the duration of exposure, the solution, containing the eggs, was poured through a 20 μm sieve and the eggs washed four times with distilled water to remove any remaining residues of the reagents. The contents collected on the sieve were then washed into 50 mL centrifuge tubes. Eggs were incubated at room temperature (with temperature ranging from 20 °C to 26 °C), using 0.1 N sulphuric acid, based on the results of the incubation tests stated above. Viability was determined after 18 days and finally confirmed after 28 days of incubation. For enumeration, an aliquot from each well-mixed sample was placed on a glass microscope slide with coverslip and a minimum of 200 eggs counted under the microscope and categorized into potentially viable or non-viable based on the criteria above. A visible egg shell without a larva or egg contents was considered as potentially viable based on the assumption that the eggs may have hatched during the course of the incubation.

Percentage viability for eggs exposed to each reagent or combination of reagents was calculated by dividing the number of viable eggs by the total number of eggs observed and multiplying by 100.

### Spiking of wastewater and sludge with A. suum eggs

Approximately 100 mL of raw untreated wastewater and 30 g of sludge were sampled from the inlet of a wastewater treatment plant for spiked exposure assessment. The stock solution containing 1,000 eggs was inoculated into containers containing the wastewater and sludge samples. For the sludge samples, 100 mL distilled water was added and mixed thoroughly before spiking. A control test was run with wastewater and sludge samples without exposure to the reagents, by spiking eggs into the respective volumes and weights of wastewater and sludge. Samples were then filtered through a 100 μm sieve, washed thoroughly with running water, into a 20 μm sieve. The process was thereafter continued as detailed in the section above.

### Statistical analysis

Descriptive analysis was undertaken to assess the mean percentage viability of eggs for each treatment (Stata, Statacorp, Texas, USA). A one tail t-test was performed to determine the statistical difference between mean percentage viability for each of the treatments used against the percentage viability as determined for the untreated A. suum eggs, as well as between eggs spiked into the different sample media. Pearson correlation analysis was done to determine the effect of duration of exposure on the viability. Data from reagents that were found to correlate negatively with longer duration of exposure was then used in regression analysis to determine the association between reagent and duration of exposure (GraphPad Software, Inc., USA). The best regression model for each treatment was chosen using the Akaike information criterion and r-squared values.

## RESULTS AND DISCUSSION

### Effect of different incubation solutions on viability of A. suum eggs

**Figure 1:**
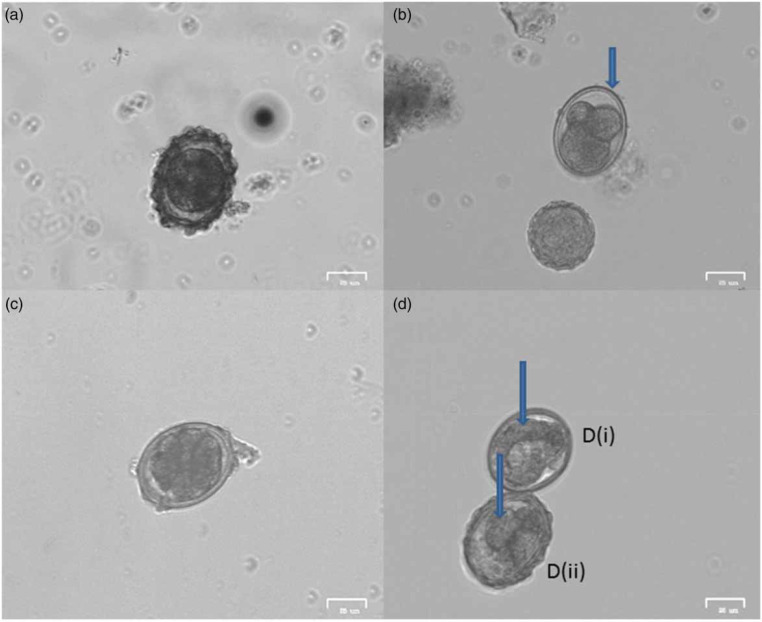
Images of potentially viable eggs at different stages of development. (a) Egg at the one cell stage; (b) decorticated egg undergoing embryonation; (c) decorticated egg at multicellular stage (>7 cells); (d) eggs with visible larvae (indicated by the arrow).

**Figure 2:**
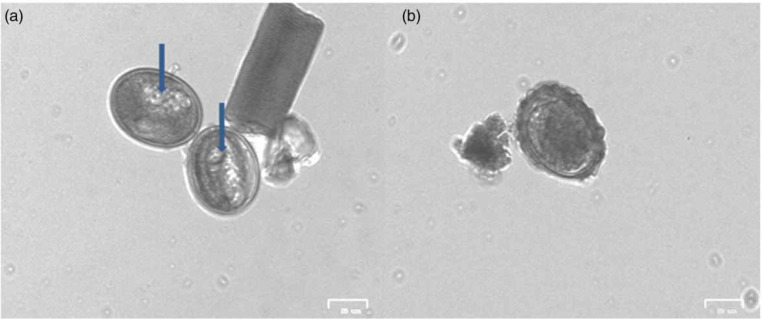
Images of non-viable eggs after incubation. (a) Decorticated eggs with visible globules (indicated by arrows); (b) dead egg.

**Figure 3:**
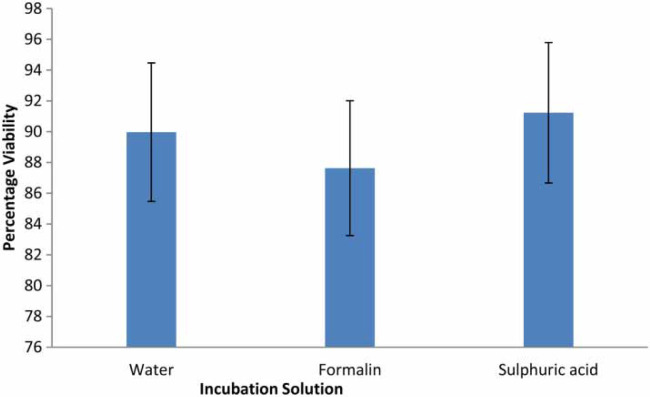
Viability of A. suum eggs with different incubation solutions (*n* ¼ 6).

Initial microscopic analysis of the eggs showed that the eggs generally were in the pre-larval stage prior to any experiment. After 28 days of incubation viable eggs were quantified based on stated criteria (‘Methodology’ section). Figures 1 and 2 show the stages of egg development. Incubation with the three solutions (distilled water, 0.5% formalin and 0.1 N sulphuric acid) gave viability of between 90.0 and 91.2% of the analysed eggs. The highest percentage of viability after the 28 days of incubation was recorded with 0.1 N sulphuric acid (91.2 ± 0.6%), followed by distilled water (90.0 ± 3.7%) and the least percentage viability was recorded with 0.5% formalin solution (87.6 ± 0.5%) (Figure 3). The difference in the percentage viabilities between these three solutions was not statistically significant at 95% confidence level (p > 0.05). These results are in line with results obtained by other researchers earlier (Nelson & Darby 2015; Karkashan *et al*. 2015). Investigations by Pecson & Nelson (2005) also showed that only 5% of A. suum eggs were inactivated during incubation in sulphuric acid. Oksanen *et al*. (1990) reported a decrease in egg viability when formalin is used as an incubation solution, which might be due to the ability of formalin to penetrate into cells by diffusion. The penetration of formalin and other chemicals into the eggs may be enhanced during hatching of the eggs, when an increase in permeability of the eggs occurs (Clarke & Perry 1988). The egg shell of Ascaris spp. has four layers, the outermost uterine layer (a glycoprotein), followed by a thin vitelline layer, a chitinous layer and then the innermost lipid layer (termed ascaroside membrane) (Quilès *et al*. 2006), which becomes permeable during hatching.

Distilled water showed a good performance as an incubation solution, although sulphuric acid gave a slightly higher percentage of viability, however not statistically significant. The results confirm the findings by Nelson & Darby (2001). Distilled water, thus, could replace other solutions that are more expensive and toxic. However, the use of distilled water as an incubation solution could also, on the other hand, result in the loss of egg viability due to growth of bacteria and fungi, which may result in egg inactivation (Ciarmela *et al*. 2002).

Sulphuric acid is very corrosive and therefore poses health concern for laboratory personnel, especially when there is direct exposure to sufficient concentrations (PHE 2015). Exposure to formalin may also result in irritation and corrosive effects (Pandey *et al*. 2000). Therefore their replacement with distilled water as an incubation solution would result in accurate viability results while ensuring health safety of laboratory personnel.

### Effect of reagents on viability of A. suum eggs spiked into distilled water

Exposure of A. suum eggs to the different reagents had effect on the viability of the egg as determined by incubation. Out of the nine reagents or combination of reagents, the highest negative effect in viability was observed due to exposure to acetoacetic acid, with only 3.4 ± 1.68% of the eggs remaining viable, followed by combination of acetoacetic acid and ethyl acetate (13.2 ± 14.4%). Ethyl acetate as well as the combination of acetoacetic acid and formalin also gave statistically significant loss in viability as compared to the mean viability determined by incubation in sulphuric acid without any treatment (Table 1). Statistically, ethyl acetate, acetoacetic acid, combination of ethyl acetate and acetoacetic acid and then the combination of acetoacetic acid and formalin resulted in mean percentage viabilities that were significantly lower than the mean percentage viability of untreated (un-exposed) eggs (Table 1). This might be due to the effect of the acid on the lipid layer of the eggs, thereby increasing permeability of the egg shell, which subsequently led to the inactivation (Clarke & Perry 1988). Although formalin and ethyl acetate did not significantly affect the viability of the eggs as individual components, their combinations with acetoacetic acid led to a significant reduction in viability. However, the individual effect of acetoacetic acid was higher. Table 1 shows that the reagents used as detergents for the dissociation of eggs from particulate matter (Tween 80 and ammonium bicarbonate) as well as solutions used for the flotation steps (magnesium sulphate and zinc sulphate) did not significantly affect the viability of the A. suum eggs. However, reagents used during the phase extraction step (ethyl acetate and acetoacetic acid) significantly affected the egg viability. These phase extraction reagents are mostly lipid-soluble and ether-absorbing chemicals and their negative effect might be due to the degradation of the lipid layer of the egg (which is the last defence for the eggs), thereby inactivating them.

**Table 1:** Detailed descriptive analysis of the mean percentage of viable eggs for each treatment (N ¼ 200)

	Ethyl acetate	Acetoacetic acid	Formalin	Ammonium bicarbonate	Zinc sulphate	Magnesium sulphate	Tween 80	Acetoacetic acid + ethyl acetate	Acetoacetic acid + formalin
Minimum	60.8	1.8	75.0	1.8	83.1	84.9	82.8	2.2	52.6
25% percentile	67.5	1.9	86.0	50.9	83.5	85.3	84.5	2.9	53.1
Median	75.9	3.3	90.6	88.1	85.9	89.1	89.0	5.6	55.5
75% percentile	80.4	4.2	95.5	90.4	94.9	90.7	90.8	29.7	68.8
Maximum	83.3	6.5	97.2	90.8	98.2	92.3	92.6	34.9	72.9
Mean (SD)	74.4 (8.3)	3.4 (1.7)	89.7 (7.8)	71.1 (35.1)	88.5 (6.3)	88.5 (2.8)	87.9 (3.6)	13.2 (14.4)	59.1 (9.3)
95% confidence interval of mean	64.1-84.7	1.6-5.2	81.5-97.8	34.2-107.9	80.7-96.4	85.5-91.5	83.4-92.4	-1.8-28.3	44.3-73.9
t-test^*^	0.0109	<0.0001	0.6878	0.2228	0.4329	0.0818	0.1343	<0.0001	0.0064

* p value < 0.05 was considered as statistically significant.

**Table 2:** Correlation between duration of exposure and the mean percentage viability

	Time	Ethyl acetate	Acetoacetic acid	Formalin	Ammonium bicarbonate	Zinc sulphate	Magnesium sulphate	Tween 80	Acetoacetic acid+ ethyl acetate	Acetoacetic acid+ ethyl formalin
Time	1									
Ethyl acetate	-0.24	1								
Acetoacetic acid	-0.47	0.54	1							
Formalin	0.15	0.86	0.19	1						
Ammonium bicarbonate	0.35	-0.04	0.24	-0.11	1					
Zinc sulphate	0.28	-0.13	0.30	-0.27	0.98	1				
Magnesium sulphate	-0.18	-0.05	0.58	-0.11	0.43	0.43	1			
Tween 80	-0.28	-0.31	0.37	-0.70	0.30	0.49	0.06	1		
Acetoacetic acid + ethyl acetate	-0.72	0.62	0.93	0.21	-0.05	0.02	0.37	0.33	1	
Acetoacetic acid + formalin	-0.61	0.43	0.80	0.05	-0.28	-0.15	0.14	0.48	0.89	1

**Table 3:** Percentage viability (± standard error) of A. suum eggs spiked into wastewater samples

Wastewater samples										
Exposure time (min)	Ethyl acetate	Acetoacetic acid	Formalin	Ammonium bicarbonate	Zinc sulphate	Magnesium sulphate	Tween 80	Acetoacetic acidþ ethyl acetate	Acetoacetic acidþformalin	
5	80.5 ± 1.5	8.5 ± 1.2	92.1 ± 6.1	92.5 ± 5.1	92.5 ± 5.2	92.1 ± 4.6	92.6 ± 6.4	56.6 ± 4.3	79.1 ± 4.9
10	81.2 ± 2.6	6.8 ± 2.0	91.3 ± 3.6	90.2 ± 5.2	91.6 ± 4.6	89.2 ± 7.6	90.1 ± 6.1	55.1 ± 3.2	64.2 ± 6.4
30	79.5 ± 1.6	6.0 ± 1.4	91.2 ± 4.5	90.6 ± 4.6	94.6 ± 7.8	88.6 ± 8.4	93.1 ± 4.2	55.9 ± 4.2	57.6 ± 5.7
60	77.6 ± 2.8	5.4 ± 1.3	90.4 ± 7.1	90.8 ± 6.5	97.8 ± 6.9	90.5 ± 6.1	90.1 ± 5.3	45.2 ± 6.2	55.6 ± 8.7
90	76.9 ± 3.6	5.5 ± 1.2	90.9 ± 5.5	89.9 ± 8.7	92.5 ± 8.4	89.6 ± 2.9	89.9 ± 6.4	40.1 ± 4.8	56.7 ± 6.7
120	76.2 ± 5.4	4.9 ± 3.1	91.5 ± 4.2	89.5 ± 9.4	97.9 ± 6.4	90.5 ± 4.6	90.1 ± 4.6	28.9 ± 7.5	54.9 ± 2.9

**Table 4:** Percentage viability (± standard error) of A. suum eggs spiked into sludge samples

Sludge									
Exposure time (min)	Ethyl acetate	Acetoacetic acid	Formalin	Ammonium bicarbonate	Zinc sulphate	Magnesium sulphate	Tween 80	Acetoacetic acid + ethyl acetate	Acetoacetic acid + formalin
5	92.6 ± 7.8	78.2 ± 3.4	93.0 ± 6.1	91.2 ± 4.3	95.6 ± 6.1	93.1 ± 2.3	92.6 ± 6.2	80.1 ± 2.3	84.2 ± 4.6
10	90.5 ± 6.5	74.6 ± 5.2	91.3 ± 3.2	91.5 ± 5.1	94.2 ± 1.6	91.2 ± 1.9	90.1 ± 6.1	68.5 ± 4.1	86.2 ± 5.1
30	89.6 ± 4.2	65.6 ± 3.2	91.6 ± 1.8	90.3 ± 2.6	93.1 ± 5.4	90.1 ± 5.2	93.1 ± 4.1	73.5 ± 2.1	72.0 ±7.1
60	88.8 ± 2.1	66.1 ± 2.8	92.1 ± 2.5	91.0 ± 4.6	92.1 ± 1.2	90.3 ± 4.2	90.1 ± 2.1	70.5 ± 3.5	63.0 ± 1.6
90	90.2 ± 3.1	64.2 ± 6.2	91.1 ± 6.1	90.1 ± 8.1	93.5 ± 3.8	90.2 ± 6.2	89.9 ± 3.1	72.3 ± 4.9	56.7 ± 2.9
120	87.6 ± 1.6	35.5 ± 1.5	90.1 ± 4.3	89.9 ± 7.0	95.6 ± 7.4	91.2 ± 7.6	90.1 ± 2.6	64.2 ± 5.6	55.1±2.4

The dose of a toxic substance that an organism is exposed to, as well as the duration of exposure to the said substance, is very important in the determination of the dose–response relationships (Nelson & Darby 2001). This is used here to evaluate the inactivation of A. suum eggs as a result of prolonged exposure to these reagents. With a fixed concentration of these reagents the duration of exposure is the main factor in the inactivation of A. suum eggs. It was found that prolonged exposure had a destructive effect on the viability of the A. suum eggs. There was a negative correlation for ethyl acetate, acetoacetic acid,

magnesium sulphate, Tween 80 and then the combination of ethyl acetate and acetoacetic acid as well as the combination of acetoacetic acid and formalin with percentage viability (shown in Table 2). Although Tween 80 and magnesium sulphate did not show any significant reduction in mean percentage viability, an increase in the duration of exposure results in a reduction in viable eggs. This makes it necessary to reduce the exposure of eggs to the reagents to the shortest time possible. The best correlation between duration of exposure and inactivation was found for the combination of acetoacetic acid and ethyl acetate, probably due to the individual effect of acetoacetic acid, as treatment with this reagent alone resulted in the highest loss of viability, with only 3.4 ± 1.68% of eggs remaining viable. This is confirmed by the correlation results presented in Table 2.

Although ethyl acetate recorded a mean percentage of viable eggs of 73.4 ± 8.3%, which was not significantlydifferent from the untreated eggs (determined during the initial screening phase with sulphuric acid, formalin and distilled water), an increase in the duration of exposure led to a greater reduction in viability. Increase in duration of exposure correlated negatively with percentage viability (0.63), which was the highest negative correlation for all the reagents studied.

This shows that although exposure for short periods (5 minutes) might not affect the viability of exposed eggs, prolonged exposure (30 minutes) might result in the loss of viability. The loss in viability might be due to the increase in permeability during the hatching of the eggs. Permeability of egg shells increases during hatching, which might be the reason for the increase in the inactivation effect of the reagents with prolonged duration (Clarke & Perry 1988). A similar relationship between prolonged exposure and percentage viability was seen for exposure to the combination of acetoacetic acid and formalin, with acetoacetic acid seen as the main contributor to the effect. This is based on the correlation between the effects of the combined treatment against treatment with the reagents individually (Table 2). Although the exposure to magnesium sulphate did not show a significant loss in viability as compared to the untreated eggs, prolonged exposure negatively correlated with the percentage of viability (0.53) which means that as duration of exposure increases there is a decrease in the percentage of eggs that retain their viability. Magnesium sulphate is used as a flotation solution in most literature as recommended by the USEPA method (USEPA 1999), but there is no maximum time of exposure recommended. In this case the duration of exposure of samples (which presumably contain the Ascaris spp. eggs or other helminths) is left to the discretion of the researcher. The results obtained indicate that with prolonged exposure there is a decrease in the number of viable eggs recovered, thereby resulting in false results. The same trend was seen for Tween 80, but its influence was very weak (0.0027). Acetoacetic acid exposure for 5 minutes (the minimum time of exposure studied) resulted in only 3.4 ± 1.7% of the eggs remaining viable and the prolonged exposure had a negative correlation with the percentage of viable eggs recovered. Based on this the use of acetoacetic acid or reagents that contain this chemical should not be recommended as it results in the loss of over 95% of viable eggs, which might result in inaccurate results if viability is determined after exposure of eggs.

**Figure 4:**
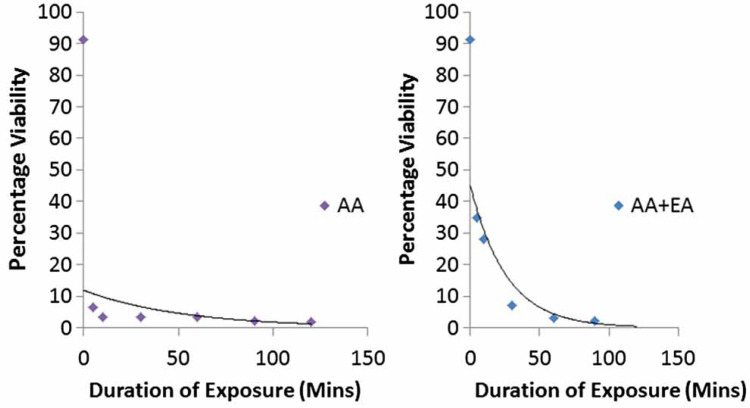
Reduction in percentage viability of eggs after prolonged exposure to treatments of acetoacetic acid (AA) and combination of acetoacetic acid and ethyl acetate (AA þ EA).

**Figure 5:**
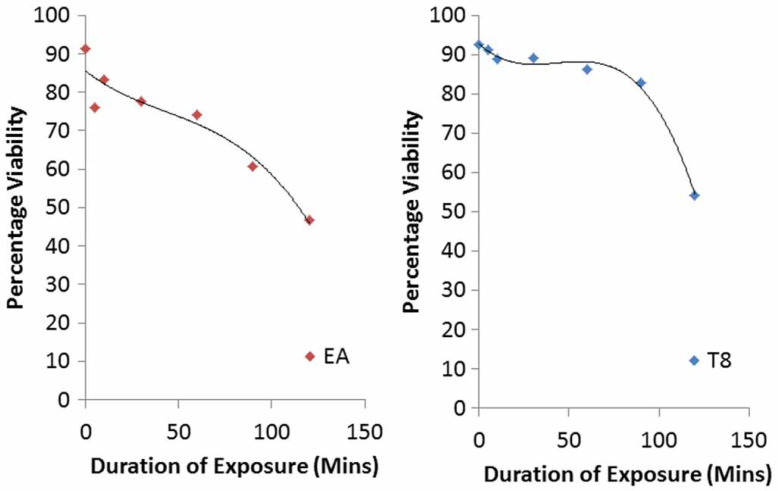
Reduction in percentage viability of eggs after prolonged exposure to treatments of ethyl acetate (EA) and Tween 80 (T8).

**Figure 6:**
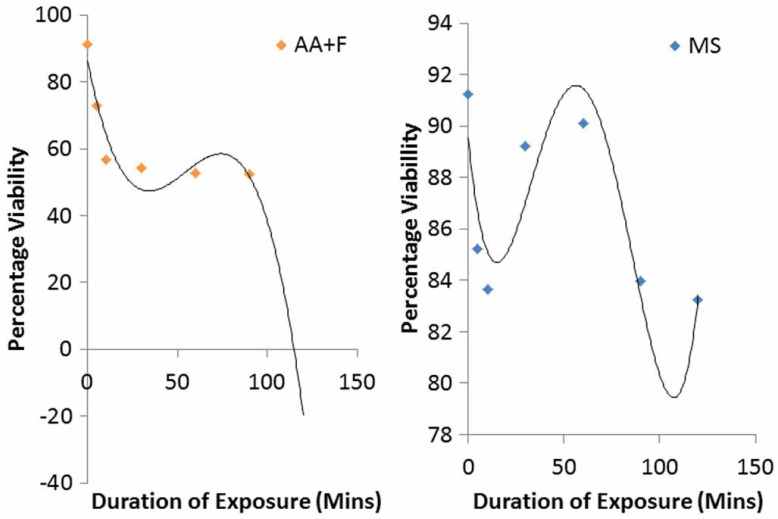
Reduction in percentage viability of eggs after prolonged exposure to treatments of magnesium sulphate (MS) and combination of acetoacetic acid and formalin (AA þ F).

For every 1 minute increase in duration of exposure, the viability of the exposed A. suum eggs reduces. The greatest decrease in percentage viability with every 1 minute increase in exposure time was for the combined exposure to acetoacetic acid and ethyl acetate (0.72), followed by exposure to acetoacetic acid (0.47), then a combined exposure to acetoacetic acid and formalin (0.61). The reduction in percentage viability for the six treatments with negative correlation between duration of exposure and reduction in percentage viability is shown in Figures 4–6.

The exponential regression model was the best fit model for treatments with acetoacetic acid and the combined treatment with acetoacetic acid and ethyl acetate (Figure 4). Exponential models are best in describing a process where the rate of an event occurring depends on the amount present (Shestopaloff 2010). Therefore inactivation of A. suum eggs with prolonged exposure to these two reagents (acetoacetic acid and the combination of acetoacetic acid and ethyl acetate) may depend on the amount of these remaining at a given time. This exponential rate of inactivation of the eggs might be due to reduction in the potency of the reagents as a result of evaporation or other unknown factors. Polynomial regression models were found to be the best fit model for treatments with ethyl acetate (cubic), magnesium sulphate (fourth order), Tween 80 (cubic), and combined treatments with acetoacetic acid and formalin (cubic). Polynomial models best predict the relationship between variables based on data present but they have poor interpolatory properties (Shestopaloff 2010). However, none of the regression models fitted for the data as explained above were statistically significant at 95% confidence level.

### Inactivation of eggs spiked into wastewater and sludge

The percentage of viability for eggs spiked into the wastewater samples as compared to the eggs spiked into the distilled water was not significantly different (at 95% confidence interval), except for exposure to acetoacetic acid (p value was 0.0001). The eggs in wastewater would thus be affected in a similar manner to eggs in the distilled water. Acetoacetic acid was found in the previous sections to result in the highest loss of viability (Table 1), but the results from the wastewater spiking tests indicate that its effect might be reduced by the properties of the wastewater (Table 3). Wastewater contains a lot of particles (Bougrier *et al*. 2005) that might have had a shielding effect against the impact of this reagent. In addition there might be components within the wastewater that may be antagonistic to the effect of acetoacetic acid. For instance, wastewater from domestic sources, as well as some industrial sources, may contain basic compounds, such as soaps and other detergents (Lajeunesse *et al*. 2008; Gracia-Lor *et al*. 2010), that react with the acetoacetic acid.

A major shielding effect was seen for eggs spiked into the sludge samples, with a higher percentage of viable eggs in these samples as compared to distilled water spiked eggs. Reagents that had a negative impact on eggs spiked in distilled water, such as ethyl acetate, acetoacetic acid, combination of these two and combination of acetoacetic acid and formalin, still resulted in the loss of egg viability but not as much as was seen in the distilled water (Table 4). The impact of these reagents was mostly severe when exposure time exceeded 30–60 minutes. After 30 minutes of exposure to acetoacetic acid, about 64.2 ± 6.1% of the eggs were still viable, which is higher than the viability of eggs in the distilled water (see Table 4).

A similar trend was seen for almost all the other reagents indicating a shielding effect. The shielding effect found in the sludge tests could be attributed to several factors; one major factor could be the presence of larger particles within the samples (Shimizu *et al*. 1997; Zorpas *et al*. 2002; Bougrier *et al*. 2005) with the potential to protect the eggs from the reagents, limiting their impact. In addition, the heterogeneity of sludge means that there may be particles within the sludge that might absorb the reagents (Hörsing *et al*. 2011; Stevens-Garmon *et al*. 2011), thereby reducing the concentration of these in the samples. A comparison of the number of viable eggs for each exposure time for eggs spiked into the wastewater and sludge showed that sludge has a much higher shielding effect than wastewater. Eggs spiked into wastewater and sludge and exposed to ethyl acetate, acetoacetic acid, combination of these two and combination of acetoacetic acid and formalin recorded significantly different percentage of viable eggs after incubation (p values <0.05). The increase in loss of viability with increase in exposure time might be due to the increase in contact between the eggs and reagents as the duration of exposure increase.

## CONCLUSION

Even though sulphuric acid had the best performance and was used in the further inactivation tests, it is important to note that the difference in percentage viability as assessed by sulphuric acid and distilled water was not statistically

significant. Distilled water performed very well (90.0 ± 3.7%) as an incubation solution, better than 0.5% formalin (87.6 ± 0.5%) which is widely used. However, there could be the loss of egg viability due to bacterial or fungal growth when distilled water is used but this effect was not determined in the current study. Therefore in conclusion distilled water may be used to reduce the cost involved in STH analysis without compromising results.

Exposure to some of the reagents used in the detection and quantification of A. suum eggs in environmental samples may affect egg viability. Acetoacetic acid was found to result in the highest loss of egg viability upon exposure; however, eggs in wastewater and sludge were shown to be least impacted. This therefore indicates that direct exposure of eggs in these samples may not result in the same magnitude of viability loss; however, the use of these reagents, as recommended by many of the methods (Ayres & Mara 1996; Schwartzbrod 1998; Collender *et al*. 2015; Amoah *et al*. 2017), is after the eggs have been concentrated from the samples and therefore the shielding effect might be lower than was seen.

Techniques that require the use of acetoacetic acid should therefore be modified to ensure that STH eggs are not inactivated. This is especially important if quantity of viable eggs is needed. The best approach would be to select methods that do not make use of this chemical.

Although most of the reagents studied did not affect egg viability within short time frames (5 minutes) of exposure, an increase in the duration of exposure adversely affected the percentage of viable eggs recovered at the end. Therefore the use of reagents such as ethyl acetate, magnesium sulphate and Tween 80 should be limited to the shortest possible time (ideally 5 minutes or less). Exposure may be reduced by thoroughly rinsing the eggs after steps involving these reagents, so as to completely remove any residual concentration of the reagents on the eggs before incubation.
